# Tricornered Kinase Regulates Synapse Development by Regulating the Levels of Wiskott-Aldrich Syndrome Protein

**DOI:** 10.1371/journal.pone.0138188

**Published:** 2015-09-22

**Authors:** Rajalaxmi Natarajan, Kara Barber, Amanda Buckley, Phillip Cho, Anuoluwapo Egbejimi, Yogesh P. Wairkar

**Affiliations:** 1 Department of Neurology and Mitchell Center for Neurodegenerative Diseases, University of Texas Medical Branch, Galveston, Texas, United States of America; 2 Summer Undergraduate Research Program, University of Texas Medical Branch, Galveston, Texas, United States of America; 3 Department of Neurology, University of Texas Medical Branch, Galveston, Texas, United States of America; 4 Neuroscience Graduate Program, University of Texas Medical Branch, Galveston, Texas, United States of America; 5 Department of Neuroscience and Cell Biology, University of Texas Medical Branch, Galveston, Texas, United States of America; Columbia University, UNITED STATES

## Abstract

Precise regulation of synapses during development is essential to ensure accurate neural connectivity and function of nervous system. Many signaling pathways, including the mTOR (mechanical Target of Rapamycin) pathway operate in neurons to maintain genetically determined number of synapses during development. mTOR, a kinase, is shared between two functionally distinct multi-protein complexes- mTORC1 and mTORC2, that act downstream of Tuberous Sclerosis Complex (TSC). We and others have suggested an important role for TSC in synapse development at the *Drosophila* neuromuscular junction (NMJ) synapses. In addition, our data suggested that the regulation of the NMJ synapse numbers in *Drosophila* largely depends on signaling via mTORC2. In the present study, we further this observation by identifying Tricornered (Trc) kinase, a serine/threonine kinase as a likely mediator of TSC signaling. trc genetically interacts with Tsc2 to regulate the number of synapses. In addition, Tsc2 and trc mutants exhibit a dramatic reduction in synaptic levels of WASP, an important regulator of actin polymerization. We show that Trc regulates the WASP levels largely, by regulating the transcription of WASP. Finally, we show that overexpression of WASP (Wiskott-Aldrich Syndrome Protein) in trc mutants can suppress the increase in the number of synapses observed in trc mutants, suggesting that WASP regulates synapses downstream of Trc. Thus, our data provide a novel insight into how Trc may regulate the genetic program that controls the number of synapses during development.

## Introduction

Synapses are the fundamental communication links between neurons and their targets. Accurate neuronal circuit function is partly determined by the number of synapses; therefore, synapse numbers are precisely regulated during development[1]. Altered synapse development is associated with some of the neurodevelopmental disorders, such as autism spectrum disorders (ASDs)[2]. Various signaling cascades act in concert to accurately establish an appropriate number of synapses. Among them, the ubiquitous mechanistic target of rapamycin (mTOR) pathway is known to play a crucial role at synapses, although its molecular mechanism of action remains to be elucidated[3].

Many patients with mutations in the tuberous sclerosis complex (*TSC*) gene suffer from various neurocognitive defects, including ASD and epilepsy[4]. Several recent studies in vertebrates have demonstrated an essential role for TSC in regulating the neuronal morphology and the function of excitatory glutamatergic synapses[5–9]. Consistent with these studies, we reported that in a fly model of TSC, loss of TSC function leads to increased number of synapses [10]. Furthermore, we suggested that mTORC2[11, 12] and not mTORC1 might be largely responsible for the increased number of synapses.

Little is known about the functionally distinct roles of mTORC1 and mTORC2, especially during synapse development. Increasingly, it is becoming apparent that mTORC2 plays important roles in neurons[13–18], including regulating synapse development and physiology [10]. Therefore, at least some neurological deficits observed in TSC patients could be due to impaired mTORC2 signaling. Taken together, these data highlight the emerging role for the mTORC2 pathway in regulating neuronal morphology and physiology during development. However, the molecular mechanisms by which mTORC2 regulates these processes remain poorly understood.

In order to understand the molecular mechanisms by which mTORC2 may regulate synapse development, especially in the context of TSC, we used a candidate screening approach and identified Trc kinase as a possible downstream genetic effector of this pathway. The tricornered (Trc) serine/threonine kinase belongs to the mammalian NDR-1 (nuclear Dbf-2 related) sub-group of AGC family of kinases and are functionally conserved from yeast to humans[19]. NDR kinases have important roles in mitotic exit, cytokinesis, proliferation, and apoptosis[19, 20], all of which require major rearrangements of the actin cytoskeleton. In sensory neurons, mTORC2 regulates Trc activity to regulate dendritic tiling [21]. A recent study hinted at the possibility that Trc kinase may function at the fly NMJs[22], but its mechanism of action and more importantly, whether it can mediate TSC signaling in neurons remains unknown.

In this study we show that *trc* mutants phenocopy the synaptic overgrowth exhibited by TSC pathway mutants and interact genetically with *Tsc2* and *rictor*- an essential component of mTORC2. Furthermore, synaptic Trc levels are significantly decreased in *Tsc2* mutants, indicating that Trc may act downstream of TSC. Interestingly, both *Tsc2* and *trc* mutants exhibited dramatic decreases in synaptic WASP levels[23]- a potent regulator of actin cytoskeleton[23, 24]. Importantly, overexpression of WASP in *trc* mutants and transheterozygotes of *trc* and *Tsc2* (which also show synaptic overgrowth) suppressed their synaptic overgrowth phenotypes. Thus, we propose that Trc kinase likely acts downstream of the TSC-mTORC2 pathway to restrict synapse numbers by regulating the synaptic WASP levels.

## Results

### Trc kinase restricts synapse growth at the *Drosophila* NMJ

Our previous study suggests that TSC restricts the number of synapses at the *Drosophila* NMJ via the mTORC2-Akt pathway[10]. To determine how this pathway regulates synapse development, we performed a candidate genetic screen.

Among the known/predicted genetic interactors of mTORC2/Akt, we chose those that are enriched in larval/adult *Drosophila* central nervous system (CNS) (S1 Table). Mutants of top ~95 candidate genes were obtained from the Bloomington Stock Center (http://flystocks.bio.indiana.edu), balanced over a green fluorescent protein (GFP) balancer to identify the homozygous mutants, and labeled with antibodies against Bruchpilot (BRP, presynaptic marker)[25], glutamate receptor (DGluRIII, postsynaptic marker)[26], and horseradish peroxidase (HRP, to mark neuronal membranes). The homozygous (non-GFP fluorescent) larvae were identified and tested for any alterations in synapse numbers. Using this strategy, we isolated three independent lines that exhibited alteration of synapse numbers at the NMJs. Of those, *arfaptin*[27] and *lim kinase*[28] are known to be required for synapse development/maintenance, and served as controls for our screen. The third candidate was Trc kinase, which had been previously implicated in regulating dendritic tiling downstream of mTORC2 in sensory neurons[18, 21]. However, several questions still remain unanswered: Does Trc function presynaptically? How does it regulate synapse development? Does it function in the TSC pathway? Before proceeding further, we first confirmed our observation that *trc* is essential for synapse development using various genetic tests as described below.

**Fig 1:**
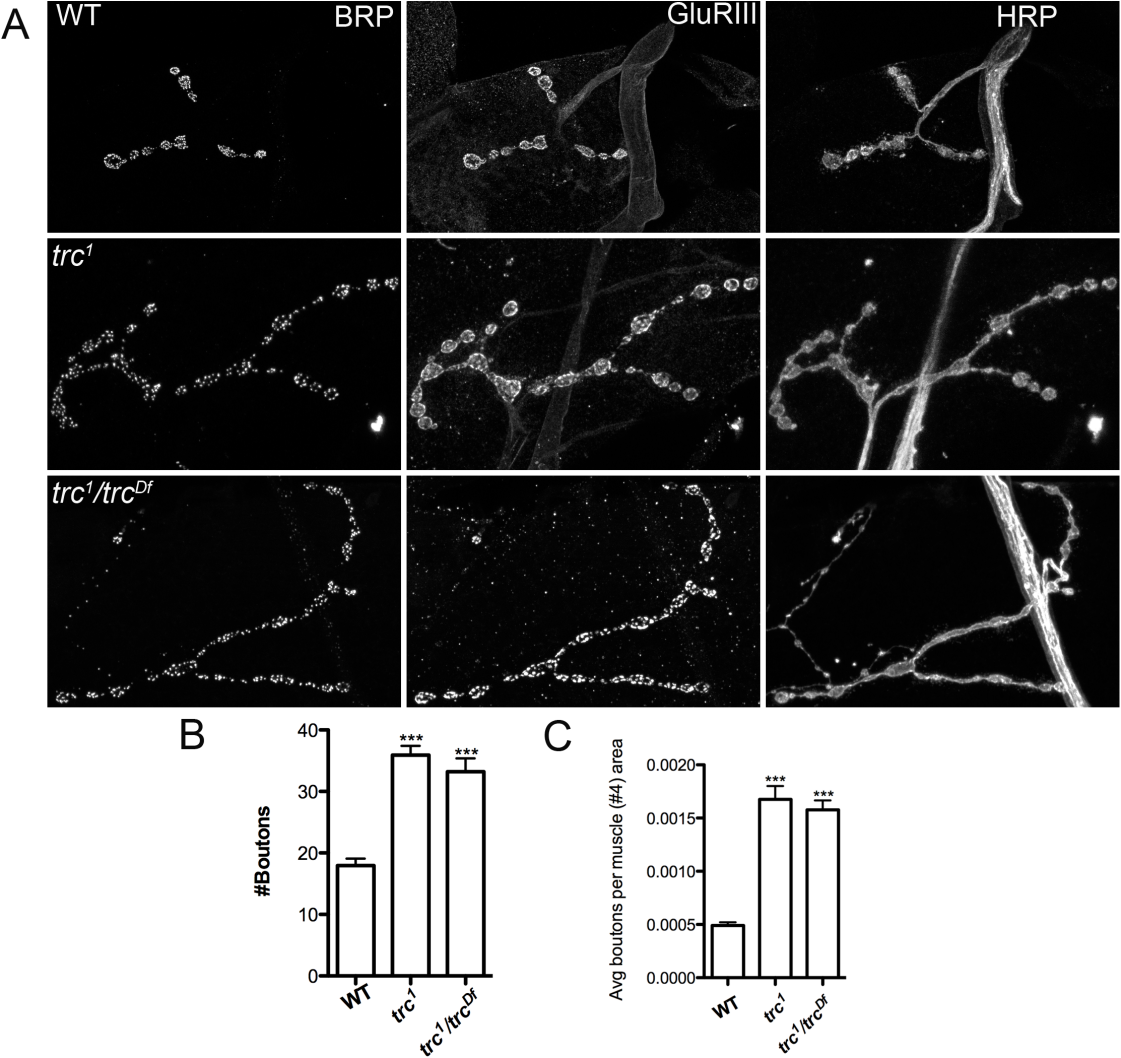
Trc kinase is required to restrict the number of synaptic boutons at the NMJ. **A**. Representative confocal stacks of muscle 4 NMJ synapses of wild type (WT), *trc* mutant (*trc* ^1^) and trc mutant crossed to an independent deficiency line (*trc* ^1^/*trc* ^Df^) that deletes the entire *trc* gene. Larval NMJs were dissected and stained with anti-Bruchpilot (anti-BRP), anti-glutamate receptor (DGluRIII) and anti-horseradish peroxidase (HRP) antibodies. **B** & **C**. Quantification of bouton numbers and bouton number per muscle area of muscle 4 from the genotypes in A. One-way ANOVA followed by Tukey’s post-hoc test was performed. n>10 and ***p<0.001. Error bars represent S.E.M.

Homozygous *trc* ^*1*^ mutants, a previously characterized loss-of-function point mutant allele[29], do not typically survive beyond the late second or early third-instar stage[22] and are also much smaller compared to age-matched wild-type (WT) larvae. This is likely because Trc regulates multiple processes that are important for survival[19]. However, upon rearing the mutant flies at 18°C we were able to find occasional escapers that barely made it to the late third-instar stage. To test its role in NMJ development, we dissected *trc* ^*1*^ mutants and labeled them with antibodies against BRP, DGluRIII, and HRP (Fig 1). Comparing *trc* ^*1*^ mutants to WT larvae revealed a significant increase in the number of synaptic boutons at the neuromuscular junctions (NMJs). However, we may have underestimated the increase because the muscle area of *trc* ^*1*^ larvae is significantly smaller than that of age-matched WT larvae (S1 Fig). Indeed, when normalized to the muscle area, *trc* ^*1*^ mutants have roughly three-fold increase in the number of synaptic boutons compared to the WT larvae (Fig 1A–1C). We also confirmed that the number of active zones (as marked by BRP) in *trc* ^1^ mutants was significantly more than the WT (S2 Fig). To confirm that loss of *trc* was responsible for the increase in synaptic boutons, we performed a genetic complementation analysis wherein homozygous *trc* ^*1*^ mutants were crossed to an independent deficiency line in which the *trc gene* (*trc* ^*Df*^) is completely deleted. The resultant progeny (*trc* ^1^/*trc* ^Df^) also exhibited an increase in the number of synaptic boutons (Fig 1A–1C), similar to the homozygous mutants. Thus, we concluded that Trc kinase is required to restrict the number of synaptic boutons at the NMJ.

Beside the increase in synaptic boutons, we also quantified other parameters of synapse growth that include, branching of axons and synaptic span. We found that there was a significant difference in the number of branches and synaptic span between *trc* mutants and WT (P<0.01) (S2 Fig). Interestingly, only synaptic span (not branching) was significantly altered in *Tsc2* mutants (S2 Fig). This suggests that Tsc2 may regulate some of the aspects of synapse growth via Trc other than the number of synaptic boutons (see below).

### *trc* acts in the *TSC* pathway to restrict synapse number

Similar increases in synaptic bouton numbers between *TSC* mutants and *trc* mutants, previous reports that mTORC2 interacts with *trc* [21]; and our previous data that suggest that TSC regulates synapse development mostly via mTORC2 [10], together suggested that Trc may function in the TSC pathway. An alternate possibility is that loss of *trc* or TSC pathway function might increase synaptic bouton numbers through independent mechanisms. To distinguish between these possibilities, we performed transheterozygous analysis between *trc* and *Tsc2/TORC2/Akt1* using the number of synaptic boutons as the readout. Unfortunately, we could not perform the double mutant analysis because single mutants of *trc* have very few larvae that can survive to the third-instar stages and the double mutant combination(s) are likely embryonic lethal because we do not find any homozygous larvae. Transheterozygous analysis is an alternate method to test if two genes affect the same pathway. We reasoned that if the transheterozygous combinations (e.g., *Tsc2/+*, *trc* ^*1*^ */+*) led to significant increases in the number of synaptic boutons, it would suggest that *trc* and *Tsc2* affect synapse growth via the same genetic pathway.

**Fig 2:**
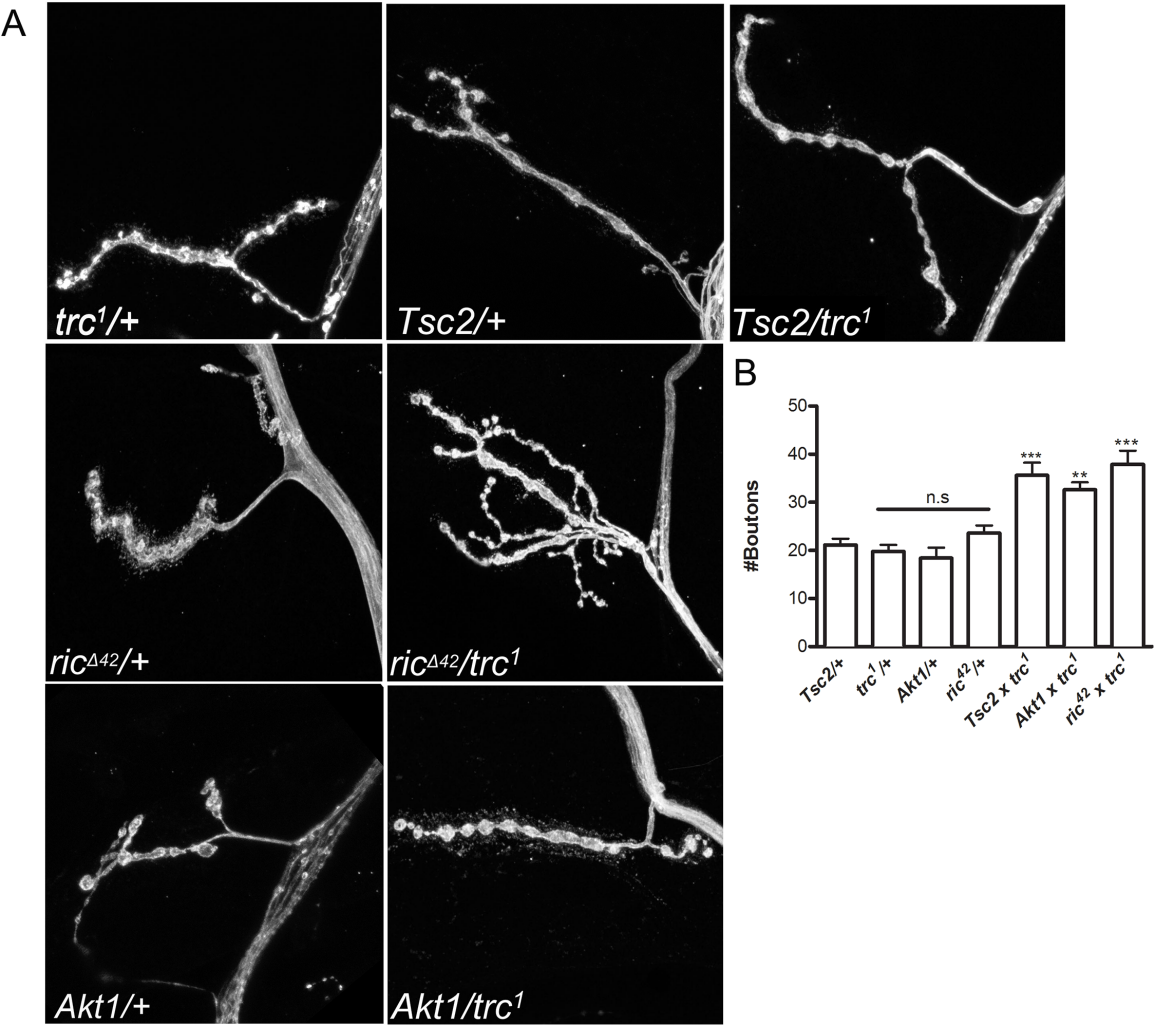
*trc* interacts genetically with *Tsc2*, *rictor* and *Akt1* to restrict the number of synaptic boutons. **A.** Representative confocal images of muscle 4 NMJ synapses of heterozygotes of *trc* ^*1*^, *Tsc2*, *rictor* ^*Δ42*^, *Akt1* marked as *trc* ^1^/+, *gig* ^109^/+, *ric* ^Δ42^/+ and *Akt1*/+ respectively and transheterozygotes of *trc* ^*1*^ with *Tsc2* (*gig* ^109^/*trc* ^1^), rictor (*ric* ^∇42^/*trc* ^1^) and Akt (*Akt1*/*trc* ^1^), stained with anti-HRP antibody. **B**) Quantification of synaptic bouton numbers from genotypes in A. n>10, One-way ANOVA followed by Tukey post-hoc test was performed. **p<0.01, ***p<0.001. Error bars represent S.E.M.

We independently crossed *trc* ^*1*^ mutants with *Tsc2 (gig* ^*109*^[30], *rictor (*mTORC2), and *Akt1* mutants respectively. Synaptic bouton numbers of the individual heterozygotes crossed to WT (Canton S (CS)) were used for comparison. We noted that unlike homozygous mutants, the transheterozygotes were comparable to the WT in overall size and muscle area (S1 Fig). As expected, heterozygous larvae of *trc* ^*1*^ (*trc* ^*1*^/+), Tsc2 (*Tsc2*/+), rictor (*ric* ^*Δ42*^/+), and Akt1 (*Akt1*/+) all showed no significant differences in synaptic bouton number when compared to the WT larvae (Fig 2A and 2B). However, transheterozygotes of *Tsc2* and *trc* ^*1*^ (*Tsc2*/+, *trc*1/+), *rictor* and *trc* ^*1*^ (*ric* ^*Δ42*^ */+*, *trc1/+*), and *Akt1* and *trc* ^*1*^ (*Akt1/+*, *trc1/+*) showed significant increases in synaptic bouton numbers, strongly suggesting that *trc* functions in the same genetic pathway as *Tsc2*, *mTORC2*, and *Akt1*. Furthermore, these interactions seemed to be specific to this pathway because *trc* did not show a transheterozygous interaction with *highwire (hiw)* [31] (S3 Fig)- one of the most potent regulators of synapse growth [32, 33]. Therefore, we concluded that *trc* might genetically interact with *Tsc2*, *mTORC2*, and *Akt1* to restrict NMJ synapse growth.

### Presynaptic but not postsynaptic Trc kinase is required to regulate NMJ synapse number

**Fig 3:**
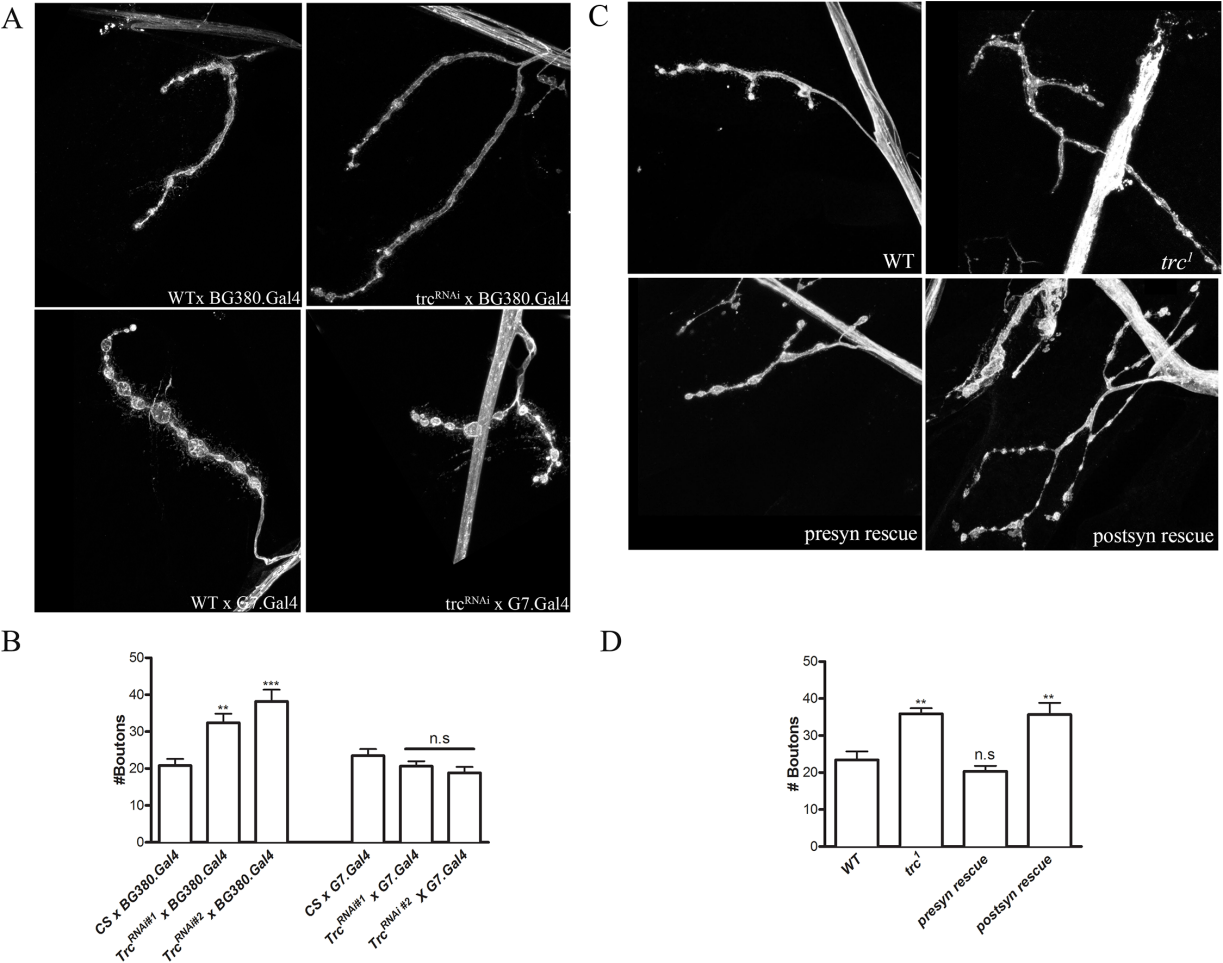
Trc acts presynaptically to regulate synaptic bouton number. **A.** Representative images of wild type (WT), *trc* ^*RNAi*^ expressed presynaptically (*trc* ^*RNAi*^ X BG380.Gal4) stained with anti-HRP antibody. Bottom Panel: Wild Type (*trc* ^*RNAi*^ X G7.Gal4) and Trc RNAi driven postsynaptically (*trc* ^*RNAi*^ X G7.Gal4) stained with anti-HRP antibody. **B.** Quantification of synaptic bouton numbers from genotypes in A. **C.** Representative confocal images from WT (Wild Type), *trc* mutant (*trc* ^1^), *trc* transgene driven presynaptically using BG380.Gal4 (Presyn rescue) and *trc* transgene driven postsynaptically using G7.Gal4 (postsyn rescue) stained with anti-HRP antibody. **D.** Quantification of synaptic bouton numbers from genotypes in C.

Our previous data indicated that Tsc2 acts presynaptically to regulate synapse growth[10]. In addition, Mob2, an interactor of *Trc* ^*1*^, can regulate synapse growth presynaptically[22]. These data suggest that the presynaptic pool of Trc may be required to regulate synapse number. To test this hypothesis, we knocked down Trc specifically in neurons or in muscles using tissue-specific Gal4 drivers[34] to drive Trc RNAi expression. Neuron- (BG380.Gal4[35]) or muscle (G7.Gal4[36])-specific drivers were used to drive the *trc* RNAi expression in presynaptic neurons or postsynaptic muscles, respectively. BG380.Gal4 or G7.Gal4 drivers crossed to WT flies served as their respective controls. Using RT-PCR, we confirmed that expression of *trc* ^RNAi^ in neurons and muscles decreased the *trc* transcripts to less than 20% of WT levels (n = 4 independent experiments with 3 larvae per genotype, p = 0.03). Presynaptic expression of two independent *trc* RNAi lines significantly increased the number of synaptic boutons (Fig 3A and 3B). On the other hand, when *trc* was knocked down in the muscles, synaptic bouton numbers were not significantly different than that of WT (Fig 3A and 3B). These data suggest that presynaptic, but not the postsynaptic, Trc kinase is likely responsible for regulating NMJ bouton numbers.

To confirm these observations, we performed rescue analyses, wherein we added back wild type Trc kinase in neurons or muscles in *trc* ^*1*^ mutants using the UAS-GAL4 system[34]. This allowed us to test if adding back the pre- or postsynaptic Trc in *trc* mutants was sufficient to restore its normal function. A full-length wild type UAS-trc transgene[37] was expressed either pre-or postsynaptically in *trc* ^*1*^ mutants. WT and *trc* ^*1*^ mutant synapses were used as controls for the comparison of synaptic bouton numbers. As expected from the RNAi experiments, presynaptic overexpression of Trc kinase restored the number of synaptic boutons close to WT bouton numbers (Fig 3C and 3D). However, postsynaptic expression of the same transgene in a *trc* ^*1*^ mutant background failed to restore the number of synaptic boutons back to WT levels (Fig 3C and 3D). We noticed that neither pre- nor postsynaptic expression of *trc* was sufficient to overcome the severe lethality or small size of *trc* ^*1*^ mutant larvae, suggesting that loss of Trc function in other tissues may contribute toward the lethality observed in *trc* ^*1*^ mutant larvae. Finally, to test whether Trc kinase is sufficient to regulate the synapse numbers, we overexpressed full-length Trc transgene presynaptically in a WT background. We did not find a significant difference between the numbers of synaptic boutons in WT larvae or WT larvae overexpressing Trc transgene presynaptically {WT = 22±3; WT with overexpression of presynaptic Trc = 23.4±2, n = 10, p = 0.4}.

To test the localization of Trc at the NMJ, we stained NMJs of WT larvae with anti-Trc antibody[38]. However, we could not detect any specific staining of Trc at the NMJ synapses. To test if Trc could localize to larval NMJs, we expressed a *trc* transgene tagged with GFP [39] presynaptically. Staining with anti-GFP antibody revealed that overexpressed Trc could localize to the NMJ synapses (S5 Fig) although this may not reflect the accurate localization of endogenous Trc. Collectively, our data suggest that presynaptic Trc is important to restrict the number of NMJ synaptic boutons.

### WASP interacts genetically with *trc* and *Tsc2*

**Fig 4:**
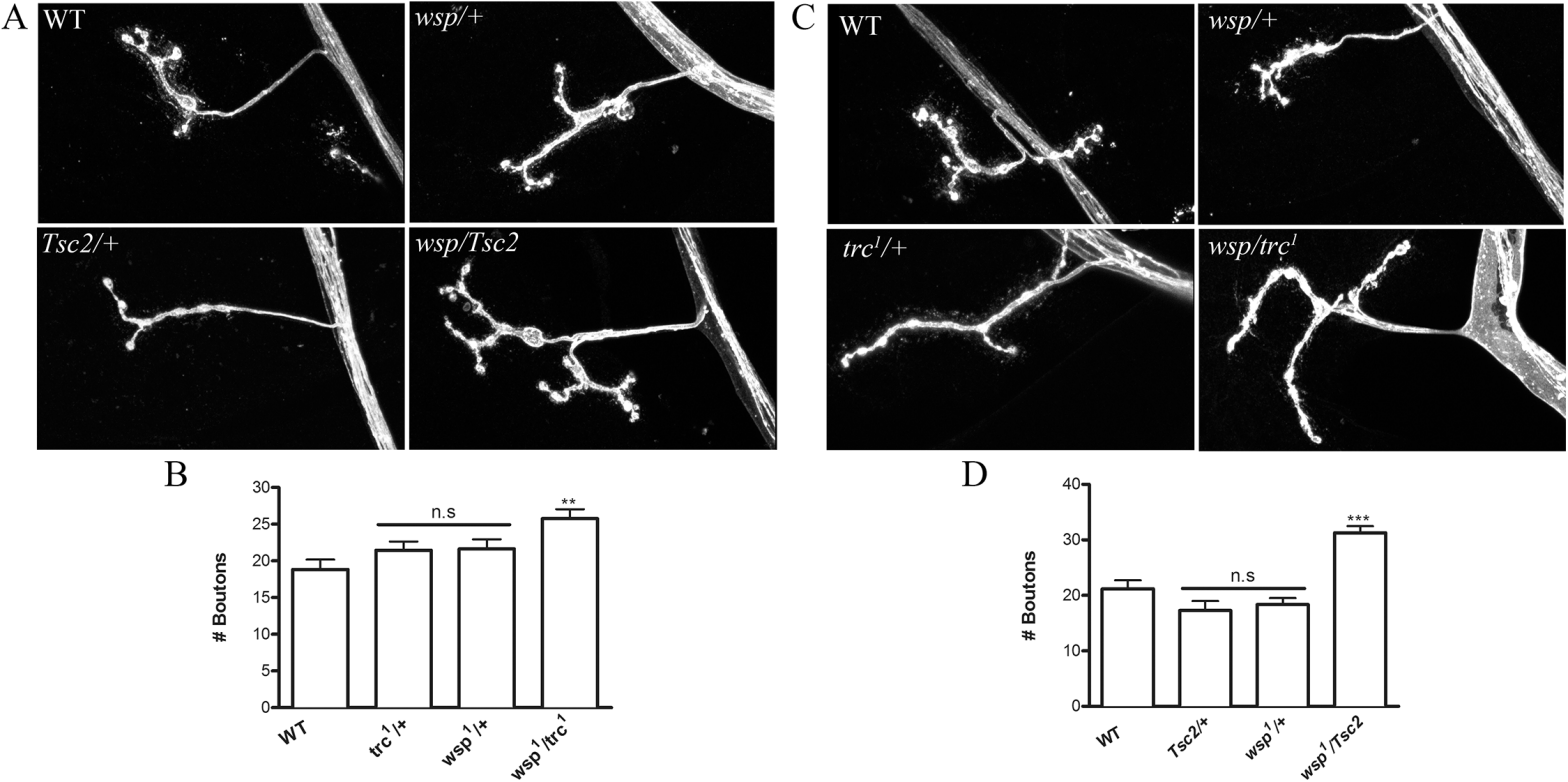
*wasp* interacts genetically with *trc* and *Tsc2*. **A**. Representative confocal images of wild type (WT), heterozygotes of *wasp* (*wsp* ^*1*^/+) stained with anti-HRP antibody. **B.** Quantification of bouton numbers in genotypes shown in A. **C.** Representative confocal images of *Tsc2* (*Tsc2*/+), *trc* ^*1*^ (*trc* ^*1*^/+) and transheterozygotes of *wasp and Tsc2* (*wsp* ^*1*^/*Tsc2*) or *wasp* and *trc* (*wsp* ^*1*^/*trc* ^*1*^) stained with anti-HRP antibody. **D.** Quantification of number of synaptic boutons in *Tsc2* and *wasp* heterozygotes (*Tsc2/+ or wsp1/+*) and their corresponding transheterozygotes (*Tsc2/wsp* ^*1*^). One-way ANOVA followed by Tukey’s post-hoc test was performed. n.s = not significant, **p< 0.01, ***p<0.001. Error bars represent S.E.M.

Trc kinase and mTORC2 control polarized outgrowths in various developmental contexts by regulating actin polymerization [19, 40, 41]. Interestingly, WASP, a potent regulator of actin polymerization [42], has also been implicated in regulating synapse development at the *Drosophila* NMJ [43–45]. In addition, akin to *trc* ^*1*^ and other *Tsc2* pathway mutants, *wasp* mutants also exhibit increased number of synaptic boutons [43, 44]. However, it is not known if WASP and Tsc2/Trc pathway act together to regulate synapse growth. To further explore the molecular mechanism of how Trc regulates synapse numbers, we asked if *wasp* interacted genetically with *Tsc2/trc* to regulate the number of synaptic boutons at NMJ. We first analyzed *trc* ^*1*^ */wasp* ^1^ and *trc* ^*1*^/*Tsc2* transheterozygotes. Corresponding heterozygotes (*Trc1/+* and *wsp* ^*1*^ */+*) and WT larvae were used as controls for comparison. As expected, a 50% decrease in *trc* ^*1*^ (*trc* ^*1*^/+), *Tsc2* (*Tsc2/+*), or *wasp* ^*1*^ (*wasp* ^*1*^ */+*) did not alter the number of synaptic boutons compared to WT. However, *Tsc2/wasp* and *Trc/wasp* double transheterozygotes exhibited significant increases in synaptic bouton numbers (Fig 4A–4D), suggesting that *wasp* interacts genetically with *Tsc2* and *trc* to restrict the growth of synaptic boutons.

### Synaptic WASP levels are reduced in *trc*, *Tsc2*, and *Akt1* mutants

**Fig 5:**
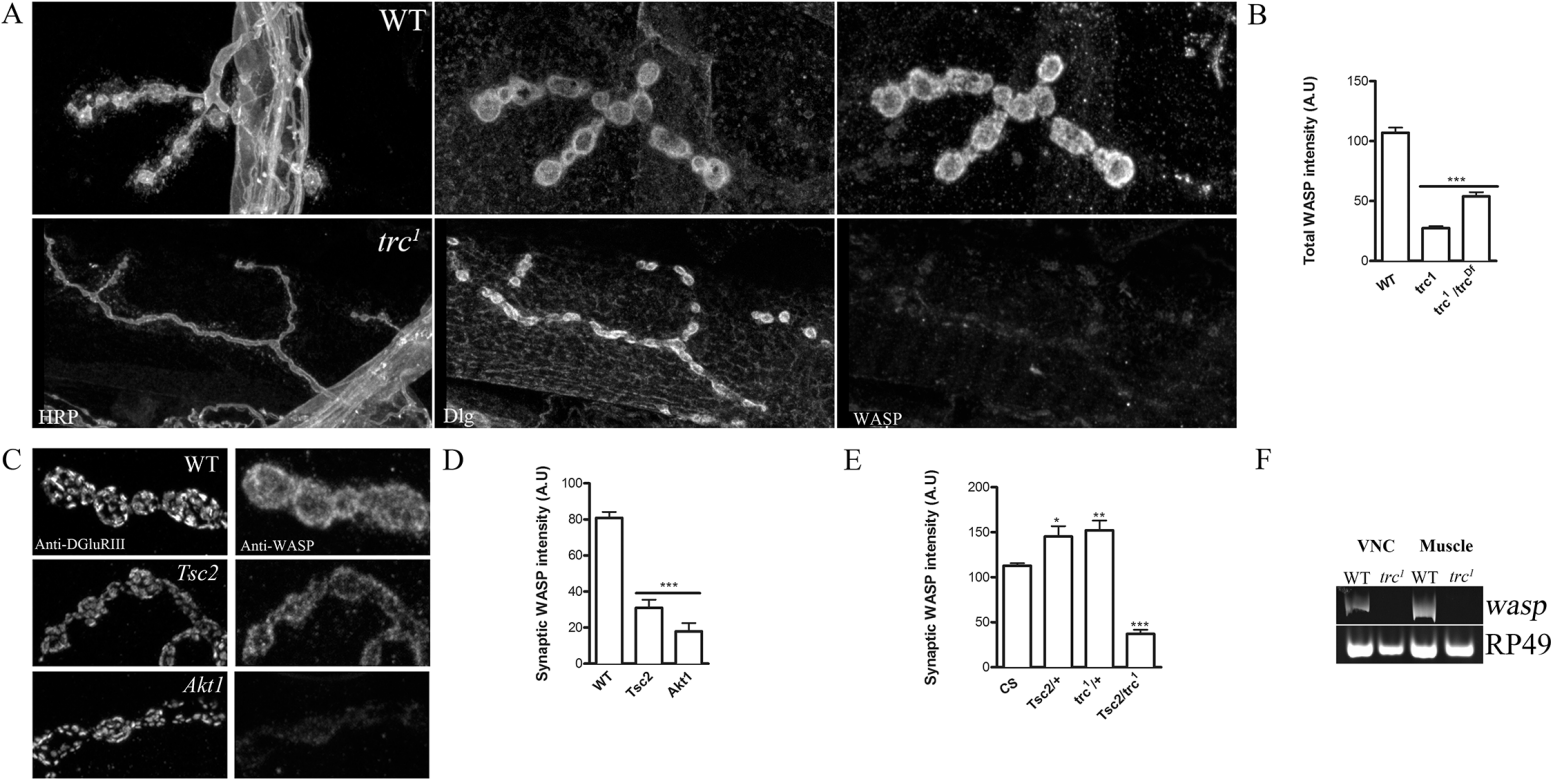
Synaptic WASP levels are reduced in *trc* and *Tsc2* mutant synaptic boutons. **A**. Representative confocal images of wild type (WT) and *trc* mutant synapses (*trc* ^*1*^) stained with anti-HRP, anti-Discs-large (Dlg) and anti-WASP antibodies. **B**. Quantification of total synaptic WASP levels in WT, *trc* ^*1*^ mutants and transheterozygote of *trc* ^*1*^ crossed to an independent deficiency line that deletes the entire *trc* gene (*trc* ^*1*^/*trc* ^*Df*^). **C**. Representative confocal images showing NMJ synapses of wild type (WT) and *Tsc2* mutants and *Akt1* mutants stained with antibodies against DGluRIII and WASP. **D**. Quantification of the synaptic WASP levels measured in genotypes described in C. **E**. Quantification of the synaptic WASP levels in WT (CS), heterozygotes of *Tsc2* and *trc* ^*1*^ (*Tsc2/+*, *trc1/+*) and in their respective transheterozygotes (*Tsc2/trc1*). One-Way ANOVA followed by Tukey’s post-hoc test was performed. *p<0.01, **p<0.001, ***p<0.0001. Error bars represent S.E.M. **F**. Representative gel showing *wasp* transcripts from ventral nerve cords (VNC) and muscles of WT and *trc* ^1^ mutants. Ribosomal protein 49 (RP49) is used as a control.

WASP is localized to NMJ synapses in *Drosophila*[43], and it was previously demonstrated that reduced synaptic WASP levels leads to an increase in synaptic boutons[44, 45]. Therefore, we hypothesized that Trc might regulate synapse growth by modulating synaptic WASP levels. To test our hypothesis, we first labeled the NMJ synapses of *trc* ^*1*^ mutants and WT larvae with antibodies against HRP, Discs large[46] (Dlg, which largely labels postsynaptic sites on muscles[47]) and WASP[48]. Consistent with previous data[43], WASP localized to pre- and post-synaptic compartments in both WT flies and *trc* ^*1*^ mutants. However, synaptic levels of WASP were dramatically reduced in *trc* ^*1*^ mutant synapses (Fig 5A and 5B) compared to WT synapses. These data suggest that Trc regulates synaptic WASP levels. Interestingly, we also found reduction in synaptic WASP in homozygous mutants of *Tsc2* and *Akt1* (Fig 5C and 5D) as well as *Tsc2/ trc* ^*1*^ transheterozygotes (Fig 5E), which is consistent with the idea that Tsc2 and Trc may regulate synaptic WASP levels to restrict synapse growth.

### Presynaptic loss of Trc kinase decreases synaptic WASP

**Fig 6:**
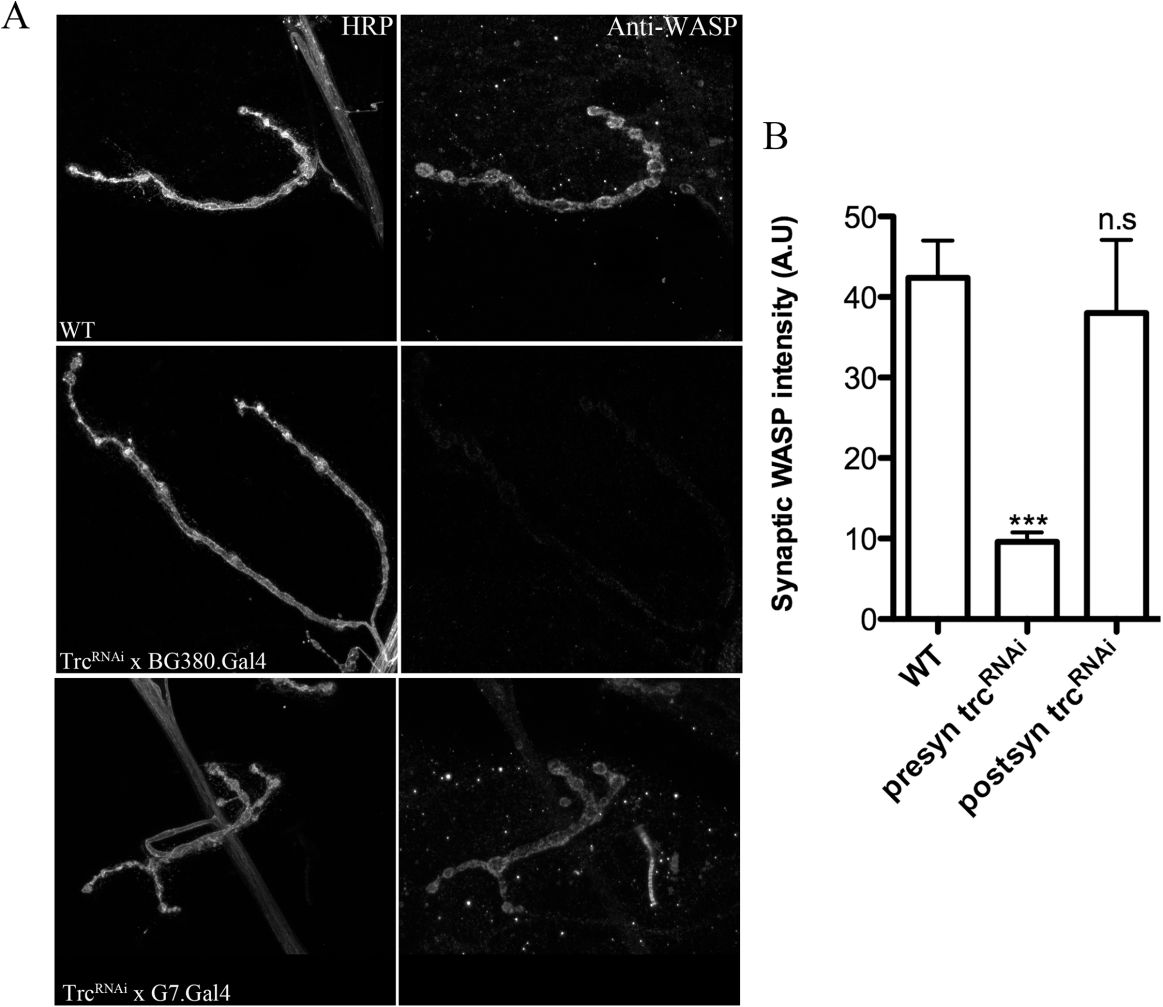
Reduction in presynaptic but not postsynaptic, Trc kinase decreases synaptic WASP levels. **A.** Representative confocal images of wild type (WT), presynaptically expressed *trc* ^*RNAi*^ (*trc* ^*RNAi*^ x BG380.Gal4) and postsynaptically expressed *trc* ^*RNAi*^ (*trc* ^*RNAi*^ x G7.Gal4) stained with anti-HRP and anti-WASP antibodies. **B**. Quantification of synaptic WASP levels in genotypes in A. n>10. One-way ANOVA plus Tukey post-hoc analysis was performed. ***p<0.001, n.s = not significant.

We next sought to test if synaptic WASP levels correlate with number of synaptic boutons. To address that question, we compared the effects of specific reduction of *trc* in presynaptic versus postsynaptic compartments because only presynaptic trc knockdown resulted in increased bouton numbers. We specifically expressed *trc* ^*RNAi*^ presynaptically using BG380.GAL4[35] or postsynaptically using G7.GAL4 driver[36]. As seen in Fig 3, presynaptic knockdown of Trc kinase increased the number of synapses and it also resulted in significant reduction in synaptic WASP levels (Fig 6A and 6B). Conversely, postsynaptic knockdown of Trc did not significantly change the number of synaptic boutons (Fig 3) or decrease synaptic WASP levels (Fig 6A and 6B). Interestingly, decrease in synaptic WASP levels might be specific to synaptic growth via Trc pathway because *hiw* mutants do not show changes in the levels of synaptic WASP (S4 Fig). These data are consistent with the idea that Trc kinase regulates synaptic bouton numbers by regulating synaptic WASP levels.

### Trc regulates the transcription of WASP

Trc kinase is a member of the Hippo pathway, whose final readout is Yorkie-mediated transcriptional regulation of genes [49]. Therefore, we wondered if altered levels of WASP in *trc* mutants could be attributed to changes in transcription of *wasp* mRNA. To compare the levels of *wasp* mRNA in *trc* mutants to WT larvae, we performed reverse transcriptase- PCR (RT-PCR). We measured the levels of *wasp* mRNA extracted from *trc* mutants or wild type larval ventral nerve cords (VNC) and the body wall, which contains most of the muscle. We used 3 larvae from each genotype per experiment and performed 4 independent experiments to compare the levels of *wasp* mRNA (Fig 5F). Compared to WT, *wasp* mRNA levels were down to less than 25% in both the VNC and less than 14% in body walls (muscle) (n = 4 independent experiments, p<0.05) of the *trc* ^1^ mutants. These data indicate that Trc kinase may regulate transcription of WASP.

### Presynaptic expression of *wsp* suppresses excess synaptic boutons in *trc* mutants

**Fig 7:**
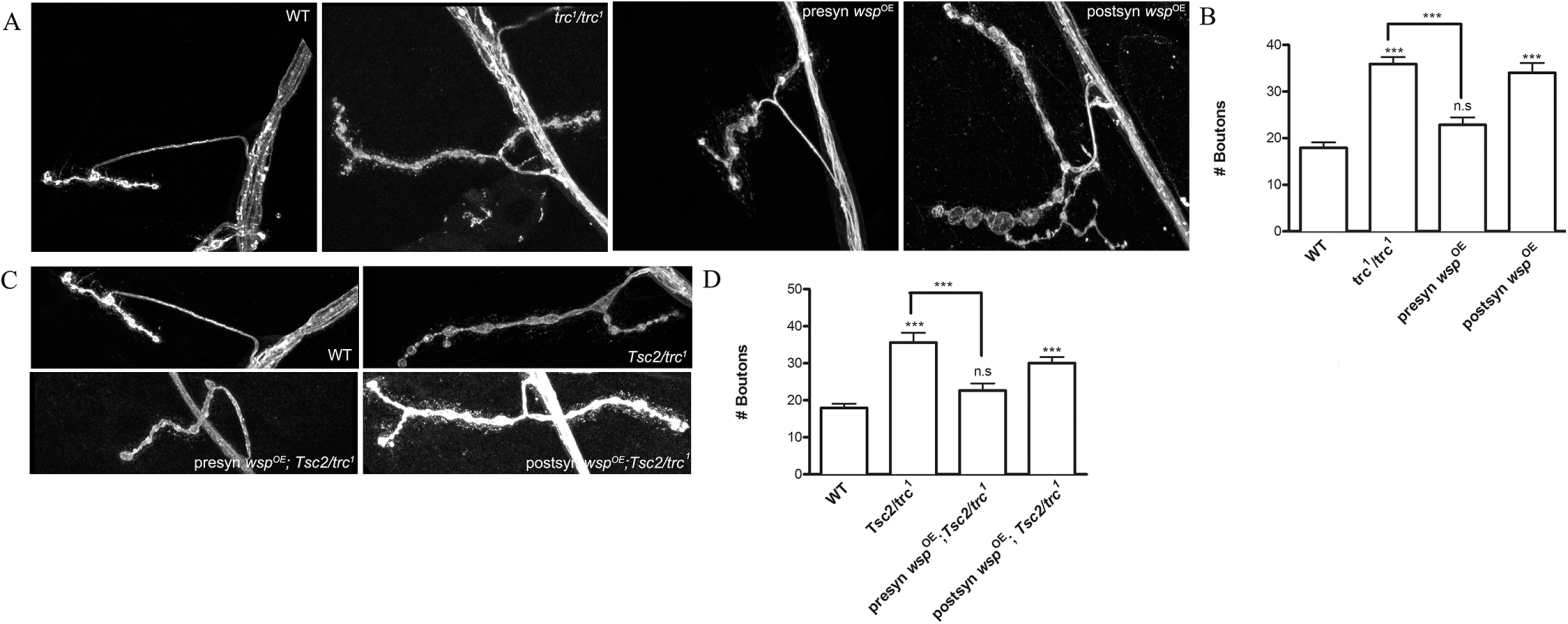
Presynaptic, but not postsynaptic, overexpression of *wasp* rescues the increase in synaptic boutons observed in *trc* ^*1*^ mutants and *trc* ^*1*^/Tsc2 transheterozygotes. **A)** Representative confocal images of the wild type (WT), *trc* ^*1*^ mutants and *trc* ^*1*^ mutants overexpressing WASP either presynaptically (presyn *wsp* ^OE^) using BG380.Gal4 or postsynaptically (postsyn *wsp* ^OE^) using G7.Gal4 driver. **B**. Quantification of genotypes in A followed by One-way ANOVA plus Tukey post-hoc test. ***p<0.001 and n.s = not significant. **C**. Representative confocal images of the wild type (WT), *Tsc2*/*trc* ^*1*^ transheterozygotes and *Tsc2*/*trc* ^*1*^ transheterozygotes overexpressing UAS-*wasp* transgene either presynaptically (presyn *wasp* ^OE^; *Tsc2/trc* ^*1*^) when crossed to BG380.Gal4 driver or postsynaptically (postsyn *wasp* ^OE^) using G7.Gal4 driver, stained by anti-HRP antibody. **D**. Quantification of bouton numbers of genotypes in C. One-way ANOVA followed by Tukey’s post-hoc test was performed. ***p<0.001 and n.s indicates “not significant”.

**Fig 8:**
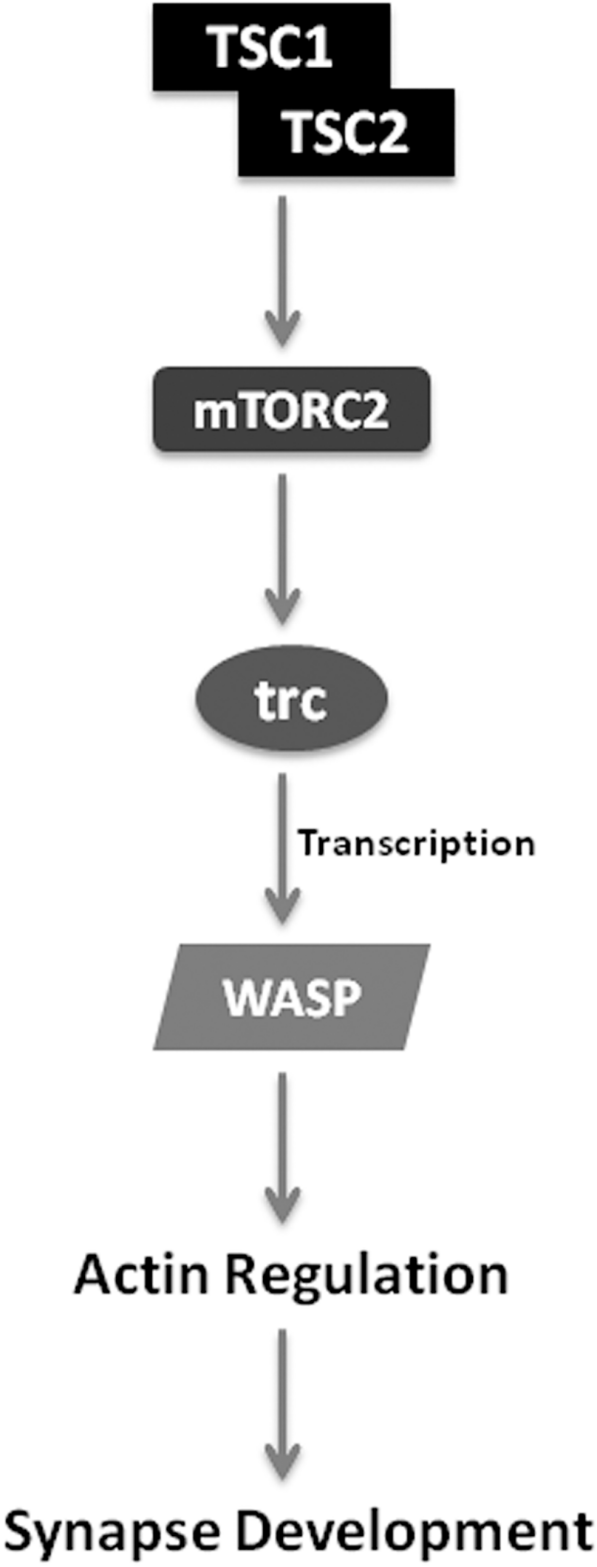
Schematic model of how Trc may regulate synapse development at *Drosophila* NMJ. Based on our data, Trc likely acts downstream of mTORC2 in the presynaptic compartment to regulate synaptic WASP, largely by regulating the transcription of WASP. Since WASP is known to regulate the polymerization of actin, we hypothesize that Trc ultimately regulates the actin cytoskeleton to regulate synapse development at the Drosophila NMJ synapses.

Previous studies have described a function for pre- and post-synaptic pools of WASP in regulating synapse development, presumably by independent mechanisms[43–45]. Loss-of-function *trc* ^*1*^ mutants and presynaptic knockdown of Trc kinase exhibit excess synapses and reduced WASP levels in both pre- and post-synaptic compartments. Therefore, we wondered whether either or both pools of WASP may be essential for synaptic growth downstream of Trc. To test if TSC-Trc pathway regulated synapse growth via pre- or post-synaptic WASP, we overexpressed *wasp* presynaptically or postsynaptically using tissue-specific Gal4 drivers in *trc* ^*1*^ mutants or in *trc* ^*1*^/*Tsc2* transheterozygotes, both of which exhibited significant increases in synaptic bouton numbers (Figs 1 and 2). We reasoned that significant reversion to WT number of boutons following pre- or postsynaptic *wasp* overexpression would indicate that WASP function was necessary to restrict synapse growth in that compartment. In addition, this would also confirm that WASP acts downstream of Tsc2 and Trc kinase. On the other hand, if both pools of WASP need to function together to restrict synapses, we expected to see negligible or partial suppression in synaptic bouton numbers following the expression of *wasp* transgene in either neurons or muscles. We observed that presynaptic *wasp* overexpression in *trc* ^*1*^ mutants led to significant decrease in the number of synaptic boutons–almost similar to that of WT (Fig 7A and 7B)–but postsynaptic *wasp* overexpression did not (Fig 7A and 7B). Similar suppression was also observed in *Tsc2*/*trc* ^*1*^ transheterozygotes (Fig 7C and 7D). Finally, there was no change in the number of boutons when *wasp* was overexpressed in an otherwise WT background {WT = 18.5 ± 2.7; Overexpression of *wasp* in WT = 20.3 ± 3.5, n = 10}. Together, these data suggest that presynaptic WASP plays an essential role in restricting synapse growth at the NMJs via the TSC-Trc pathway (Fig 8).

## Discussion

Synapse development is a highly coordinated process that is precisely programmed and regulated by various signaling pathways. Dysregulation of these processes leads to alterations in synaptic function and can result in cognitive impairments, including ASDs[2, 50]. TSC signals via the mTOR kinase to regulate various aspects of neuronal morphogenesis, including proliferation, autophagy and synapse development [5, 10, 11, 51]. However, the precise mechanisms by which the TSC-mTOR pathway regulates synapses remains to be elucidated. In this study, we identified a novel role for Trc kinase, a member of the AGC family kinases[20], in the TSC-mediated growth of NMJ synapses. Although a previous study suggested that Trc kinase has a role in synapse regulation at the NMJs[22], its molecular mode of action remains unknown. In this report we demonstrate that Trc acts downstream of TSC-mTORC2 pathway. Moreover, we demonstrate that Trc restricts synaptic bouton numbers likely by decreasing the levels of presynaptic WASP, a known regulator of actin polymerization[42].

### Trc kinase acts downstream of TSC-mTOR pathway

Recent studies have shown that mTORC2 can regulate Trc kinase in sensory neurons[18, 52, 53]. Here, we suggest a novel functional link between the TSC-mTORC2 pathway and Trc kinase for the proper development of NMJ synapses. We demonstrate that Tsc2-mTORC2 largely acts through Trc kinase to regulate the levels of presynaptic WASP, presumably by affecting actin polymerization.

Trc/NDR1 kinases have been implicated in regulating polarized outgrowths in various developmental contexts, including budding in yeast, morphogenesis of sensory bristles, and dendritic patterning in *Drosophila*[19]. While the mechanistic details of how NDR1 kinases act in different developmental contexts are not clear, it is known that Hippo and Furry regulate dendritic tiling pattern establishment in sensory neurons. Interestingly, neither *hpo*[54] nor *fry*[21] mutants exhibit the synaptic overgrowth phenotype that we observe in *trc* mutants (Natarajan and Wairkar, S1 Table and unpublished data). This is consistent with our hypothesis that non-canonical upstream activators and/or signaling pathways (TSC/mTOR) may regulate Trc kinase at NMJs.

Interestingly, a recent study identified a role for *mob2*, a gene encoding an NDR kinase activator, in NMJ development[22]. Furthermore, it has been demonstrated that Mob2 physically interacts with Trc kinase in *Drosophila* epidermal hairs during development[38]. So, it is possible that Mob2 rather than Fry is the activator of Trc at the NMJ synapses. Consistent with this idea, our data and a previous report^14^ suggest that both Mob2 and Trc act presynaptically to regulate synapse growth. It would be interesting to test if Mob2 functions in the TSC pathway and whether Trc kinase can be directly activated by mTORC2, similar to its role in dendrite development[18].

### Trans-synaptic regulation of WASP by Trc

Actin cytoskeleton is important in regulating synapse development, as well as for the formation and storage of long-term memory[55]. A recent study suggests that mTORC2 might play a role in regulating long-term potentiation via regulation of the actin cytoskeleton[16]. However, mechanism by which mTORC2 regulates actin cytoskeleton remains unknown. Our data suggest that Tsc2 somehow activates Trc kinase, which in turn, regulates the synaptic levels of the actin polymerization protein, WASP. Interestingly, presynaptic knockdown of Trc caused a significant decrease in total synaptic WASP levels and increased synapse numbers while postsynaptic WASP knockdown did not. Since WASP is present both presynaptically and postsynaptically, this raises an interesting possibility that Trc may regulate the levels of WASP trans-synaptically. One of the best-studied anterograde pathways know to function at the fly NMJ synapse is the Wnt/wingless signaling pathway[56]. Indeed, there are instances where the TSC pathway has been shown to interact with Wnt/wingless signaling pathway[57, 58]. Intriguingly, in at least in one of those cases, Tsc2 is known to act upstream of Wnt signaling pathway[57].

### Role for Trc in muscle

While experimental evidence suggests that WASP may regulate synapses both pre-[43, 44] and postsynaptically[45], our data suggest that Trc only requires presynaptic WASP to regulate fly NMJ synapses. So what might be the role for WASP in the muscles? Interestingly, studies in *Drosophila* and mice have shown that Neuronal-WASP (N-WASP) and Arp2/3-mediated actin polymerization are essential for myoblast fusion during myogenesis[59–61]. Therefore, we speculate that perhaps the increased fragility of muscles that we observed in *trc* mutants is due to a reduction in postsynaptic WASP levels. Given the significantly smaller (S1 Fig) and extremely fragile muscles in *trc* ^*1*^ mutants, it is possible that postsynaptic Trc kinase may have an important hitherto unidentified function in the development of musculature.

### How does Trc regulate WASP levels?

Trc could regulate WASP levels in the following ways: by affecting its transcription, translation or by influencing the overall stability or localization of WASP. The last alternative is less likely because although we observed a decrease in WASP levels, we did not find any evidence of gross mislocalization of WASP protein at the NMJ synapses.

So, how does Trc regulate WASP levels? Trc is a member of the Hippo pathway, which regulates processes such as cell growth via transcription of downstream targets[49]. It does so via a protein called Yorkie (YAP in vertebrates), which upon activation, translocates to the nucleus to initiate specific transcription programs[49].

Our data shows that *wasp* transcripts are significantly decreased in *trc* mutants as compared to the WT, suggesting that Trc may regulate a transcriptional program for the regulation of WASP levels. Further studies are needed to test whether this regulation is achieved in a Yorkie-dependent manner or via other independent mechanisms.

### Upstream modulators and downstream effectors of Trc in neurons

Our data suggest that neuronal Trc can be regulated by Tsc2 to regulate synapse development via WASP. Phosphatidylinositol 4,5-bisphosphate (PIP_2_) and nervous wreck (Nwk) are other effectors that are known to influence synapse development via WASP. Both of them are thought to regulate presynaptic WASP leading to alterations in NMJ growth[43, 44]. Therefore, we asked if these proteins act via the Tsc2 pathway. Our preliminary data does not show any transheterozygous genetic interaction between Trc and Nwk or Tweek [62], an upstream regulator of PIP_2_ (Natarajan and Wairkar, unpublished data), indicating that Nwk/PIP2 affect synapse growth independent of Tsc2-Trc pathway. However, absence of transheterozygous interactions are not conclusive and therefore, we cannot completely rule out an interaction between these genes. Further analysis is needed to confirm whether Nwk and Tweek interact with Trc.

What are the downstream targets of Trc? A recent chemical genetic screen, which sought to identify downstream regulators of Trc in dendrites, found that the mammalian homolog of Trc (NDR-1 kinase) regulates dendritic arborization and excitatory synaptic function via its substrates AAK1 (AP-2 associated kinase) and Rabin8, a GTP/GDP exchange factor of Rab8[53]. Interestingly, both these substrates appear to regulate distinct aspects of Trc-mediated dendritic growth- AAK1 is important for the regulation of dendritic branching, and Rabin8 is critical for spine morphogeneis. Our data add to this information and suggest that WASP is another potential downstream mediator of Trc signaling pathway, with perhaps, a specific role in synapse development.

### Potential role of Trc kinase in neurodevelopmental disorders

The loss of Trc leads to abnormal dendritic tiling in flies, and it has also been implicated in dendrite maintenance[21]. These functions of Trc seem to be conserved in mice because loss of NDR1 kinase leads to defects in spine morphogenesis and dendritic arborization[53], which are reminiscent of some neurodevelopmental disorders[63–65]. These data support the idea that abnormal synapse development might be one of the major contributors to neurodevelopmental disorders. Previous studies[18, 21, 53, 66] and our data support this hypothesis. We found that Trc plays a role in synapse development downstream of Tsc2, which has been strongly implicated in Autism Spectrum disorders [67, 68]. Thus, a growing body of evidence indicates that Trc/NDR1 kinase signaling maybe important in neurodevelopment, and future studies are likely to address the precise molecular mechanisms that regulate Trc kinase and its role in neurodevelopmental disorders.

## Materials and Methods

### Fly stocks

All fly lines were reared in medium containing Nutri-Fly^TM^ Bloomington formulation (Genesee Scientific, San Diego, CA), Jazz mix (Fisher Scientific, Waltham, MA, USA), sugar and powdered yeast (Genesee Scientific) in an 8:5:1:1 ratio and made according to standard procedures. To allow for the survival of homozygous *trc* ^*1*^ mutant larvae up to the early third-instar stage, flies had to be reared in bottles containing the medium described above at 18°C. We ensured that the bottles were optimally populated (~50–70 flies/bottle (max)). WT flies were either Canton S (CS) or CS outcrossed to *w* ^*−*^, BG380.Gal4[35] or G7.Gal4[36] lines depending on the experiment. The following fly lines were obtained from the Bloomington Stock Center: *trc* ^*1*^[38], *trc* ^*Df*^: *Df*[3L]*BSC445*, *wsp* ^*1*^ *and UAS-Wasp*[48], *scar* ^*1*^, *gig* ^*109*^[30] (*Tsc2*), *Akt1* ^*04226*^[69], and *rictor* ^*Δ42*^[70]. Expression of transgenic *trc* ^RNAi^ lines: *trc* ^*GL01127*^ P{TRiP.GL01127} [71] and *trc* ^*JF02961*^ P{TRiP.JF02961}[72] or p(UAS-trc-GFP)[41] was driven using the Gal4 lines mentioned above. BG380.Gal4 was obtained from Aaron DiAntonio, Washington University Medical School (St. Louis, MO, USA).

### Immunohistochemistry

Larvae were dissected and labeled as described previously[26] using rabbit anti-DGluRIII[26] (1:1000) and mouse anti-BRP[25] (1:250) primary antibodies (both from Developmental Studies Hybridoma Bank, Iowa City, IA). We also used mouse anti-Dlg (1:2000) (mAb 4f3), developed by Corey S. Goodman (Renovis, San Francisco, CA, USA) and obtained from the Hybridoma Bank; guinea pig anti-WASP[48] (1:1000), a gift from Dr. Scherzer (Rehovolt, Israel). Goat Cy5/dylight anti-HRP antibody (1:1000), Cy3- and mouse or rabbit Alexa 488- or Cy3- conjugated secondary antibodies and Alexa 633 or Cy5-conjugated anti-guinea pig antibody (1:1000) were obtained from Jackson ImmunoResearch (West Grove, PA, USA).

### Imaging and analysis

All the NMJ imaging was done at muscle 4, segment A2–A4. Confocal imaging and analysis were performed as described previously[26]. Bouton numbers were manually counted and normalized to the muscle size. Although bouton quantifications for muscle 4 are shown in the figures, synaptic overgrowth was apparent and was qualitatively similar at all muscles observed, including muscle 6/7. NMJ labeling intensities were quantified with MetaMorph software (Molecular Devices, Sunnyvale, CA, USA). Student's *t*-tests were used to compare two samples, and one-way analyses of variance (ANOVAs) followed by Dunnett's or Tukey's multiple comparison tests were performed to identify statistically significant differences within a group.

### RT-PCR Experiments

RNA was extracted using Trizol (Ambion, Life Technology) from wandering third instar larvae (3 larvae per sample, each experiment repeated four times) according to the manufacturer’s instructions. For semi quantitative RT-PCR, 1μg of pure RNA of each genotype was reverse transcribed using Superscript III RT kit (invitrogen) primed with oligo dT. cDNA thus obtained was amplified using the following primers:

*wasp* Forward Primer: 5’-TGA TGG TCA TGT GGG ACT AAA C-3’

*wasp* Reverse Primer: 5’-GCA GTT CTT TGG TTG CCA TTA G-3’

RP49 Forward Primer: 5’ CGG ATC GAT ATG CTA AGC TGT 3’

RP49 Reverse Primer: 5’ GCG CTT GTT CGA TCC GTA 3’

Trc Forward Primer: 5’ ACT ACA GTT TCC AGG ATG CCG TC 3’

Trc Reverse Primer: 5’ CCT GCG ACA AGT CCC GAT AA 3’

## Supporting Information

S1 Fig *trc* ^*1*^ mutants have reduced muscle area. **A**) Graphical representation of the decrease in muscle #4 area of *trc* ^*1*^, *and trc* ^*1*^ */trc* ^*Df*^ compared to that of wildtype **** p<0.0001. **B**) Graphical representation of muscle #4 area of wild-type (WT), heterozygotes of *trc* ^*1*^ (*CS* x *trc1*), *Tsc2* (*CS* x *Tsc2*), *Akt1* (*CS* x *Akt1*), *ric* ^*Δ42*^ (CS x *ric* ^*Δ42*^ *)* and transheterozygotes of *Tsc2/trc* ^*1*^ (*Tsc2 x trc* ^*1*^) or *Akt1/trc* ^*1*^ (*Akt1* x *trc* ^*1*^). n>10. n.s = not significant. For A and B, One-way ANOVA with Tukey post-hoc test was performed. Error bars represent S.E.M.
(TIF)

S2 Fig Other parameters of synapse growth. **A**. Bar graph representing number of active zones (as measured by BRP puncta count) at muscle 4 of WT and *trc* ^1^ mutant larval NMJs. **B & C**. Quantification of synaptic branch points (**B)** and synaptic span **(C)** in WT, *trc* ^1^, and *Tsc2* mutant (*gig* ^109^).
(TIF)

S3 Fig *Highwire* mutant does not show a transheterozygous interaction with *trc*. **A.** Representative confocal images stained with anti-Hrp (Red) and anti-Dlg (Green) antibody from muscle 4. The genotypes are as follows: WT crossed to hiw mutant (*hiw* ^Δn^), *hiw* ^Δn^ X *trc* ^1^ and *hiw* ^Δn^. **B**. Quantification of synaptic boutons from the identical genotypes as in A. n = 15, p<0.01. One-way ANOVA with Tukey post-hoc test was performed. Error bars represent S.E.M.
(TIF)

S4 Fig *Highwire* mutant does not show a change in the levels of synaptic WASP. **A**. Representative NMJ synapses from WT and *hiw* (*hiw* ^ΔN^) stained using antibodies against HRP and WASP. **B**. Quantification of levels (Intensity) of synaptic WASP in WT and *hiw* mutants.
(TIF)

S5 Fig Overexpressed Trc can localize to NMJ synapses. Representative images from muscle 4 of WT and flies overexpressing GFP-tagged Trc when expressed using neuronal driver (BG.380 GAL4). The preparation was stained using antibodies against GFP and HRP.
(TIF)

S1 File Supplementary Methods. (DOCX)

S1 Table Candidate screen of potential interactors of mTORC2/Akt. Table showing the genes that are thought to play a role in mTORC2/Akt pathway. The first column represents the *Drosophila* homolog screened and the mammalian homolog is in parenthesis. Second column represents the alleles screened in our screen and the third column reports the results from the screen. If the NMJs were altered in any way (more or fewer synaptic boutons) then it is represented in the column as “Yes” or else it is referred to as “No”.
(PDF)
