# Supplementary figures and images for: Tricornered Kinase Regulates Synapse Development by Regulating the Levels of Wiskott-Aldrich Syndrome Protein
Source: PLoS One. 2015 Sep 22;10(9):e0138188. doi: 10.1371/journal.pone.0138188 (PMC4578898; doi:10.1371/journal.pone.0138188)

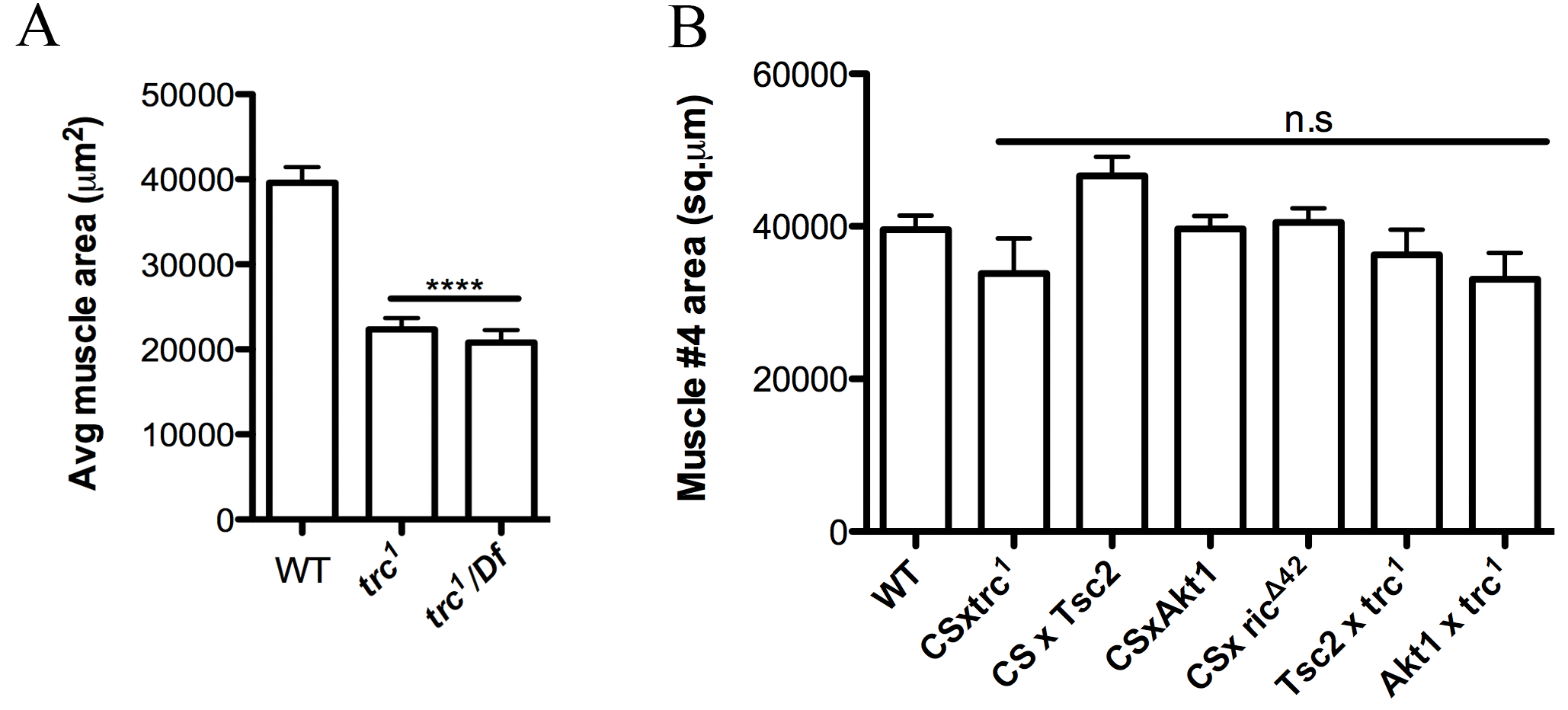

Supplement: S1 Fig — A) Graphical representation of the decrease in muscle #4 area of trc 1, and trc 1 /trc Df compared to that of wildtype **** p<0.0001. B) Graphical representation of muscle #4 area of wild-type (WT), heterozygotes of trc 1 (CS x trc1), Tsc2 (CS x Tsc2), Akt1 (CS x Akt1), ric Δ42 (CS x ric Δ42 ) and transheterozygotes of Tsc2/trc 1 (Tsc2 x trc 1) or Akt1/trc 1 (Akt1 x trc 1). n>10. n.s = not significant. For A and B, One-way ANOVA with Tukey post-hoc test was performed. Error bars represent S.E.M. (TIF) [file pone.0138188.s001.tif]

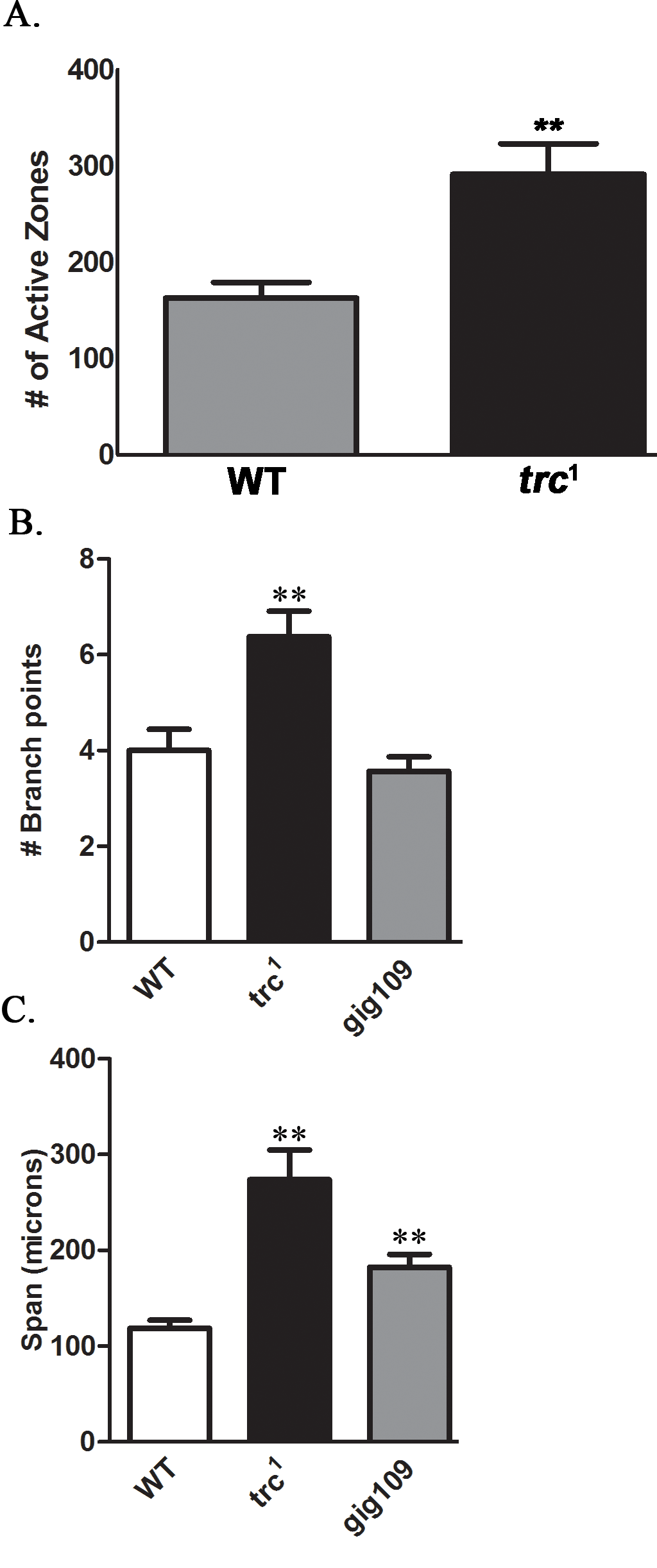

Supplement: S2 Fig — A. Bar graph representing number of active zones (as measured by BRP puncta count) at muscle 4 of WT and trc 1 mutant larval NMJs. B & C. Quantification of synaptic branch points (B) and synaptic span (C) in WT, trc 1, and Tsc2 mutant (gig 109). (TIF) [file pone.0138188.s002.tif]

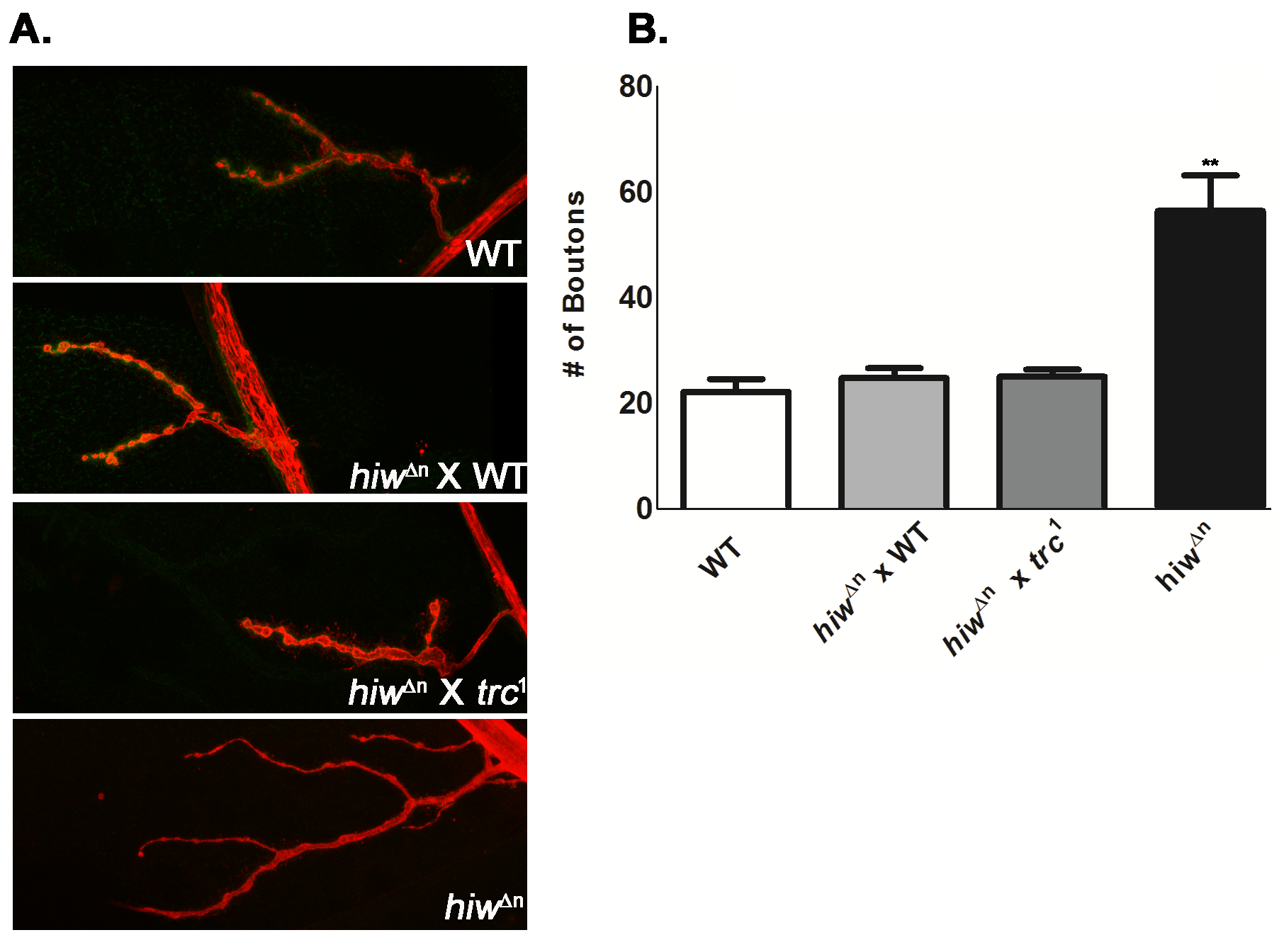

Supplement: S3 Fig — A. Representative confocal images stained with anti-Hrp (Red) and anti-Dlg (Green) antibody from muscle 4. The genotypes are as follows: WT crossed to hiw mutant (hiw Δn), hiw Δn X trc 1 and hiw Δn. B. Quantification of synaptic boutons from the identical genotypes as in A. n = 15, p<0.01. One-way ANOVA with Tukey post-hoc test was performed. Error bars represent S.E.M. (TIF) [file pone.0138188.s003.tif]

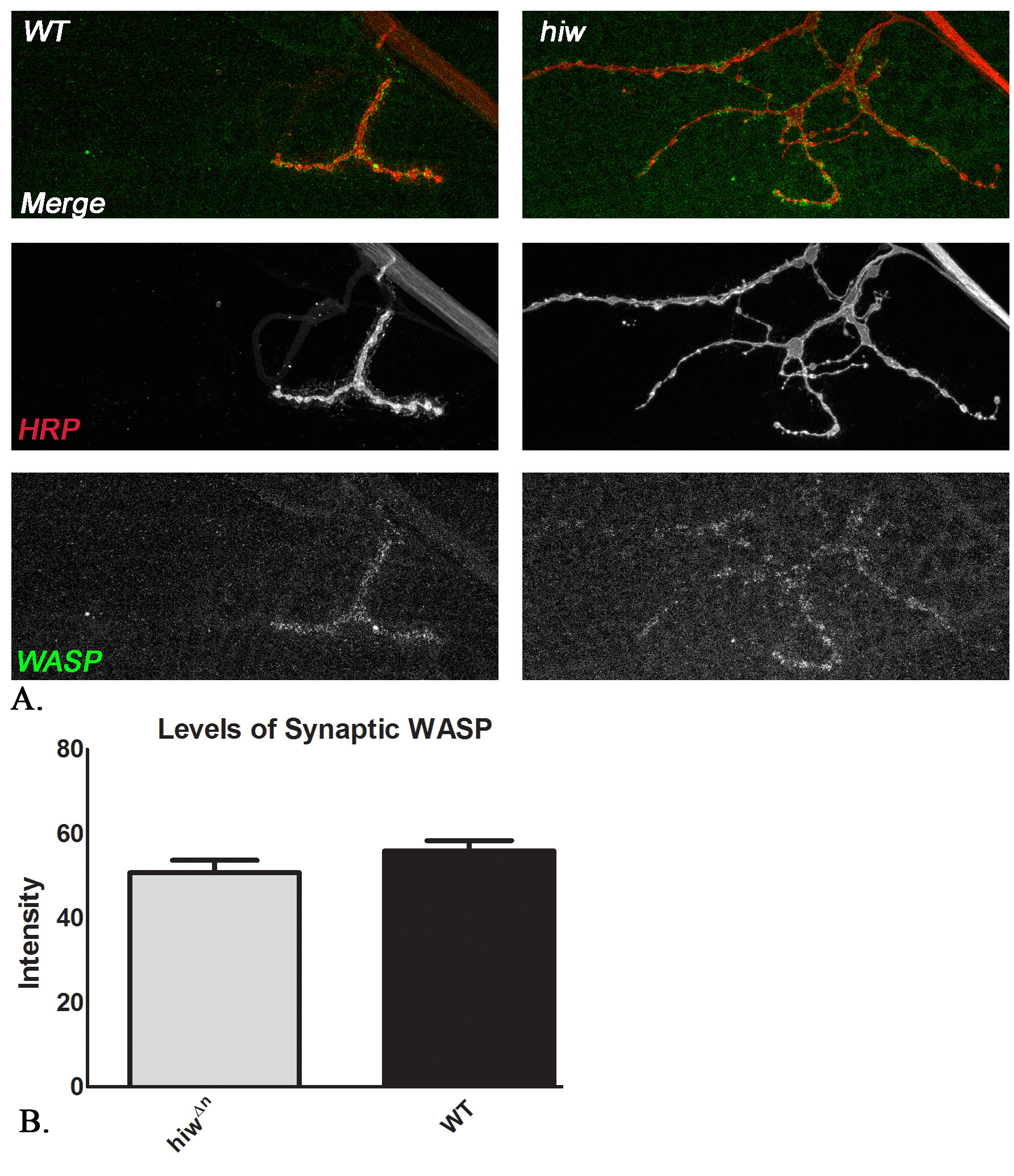

Supplement: S4 Fig — A. Representative NMJ synapses from WT and hiw (hiw ΔN) stained using antibodies against HRP and WASP. B. Quantification of levels (Intensity) of synaptic WASP in WT and hiw mutants. (TIF) [file pone.0138188.s004.tif]

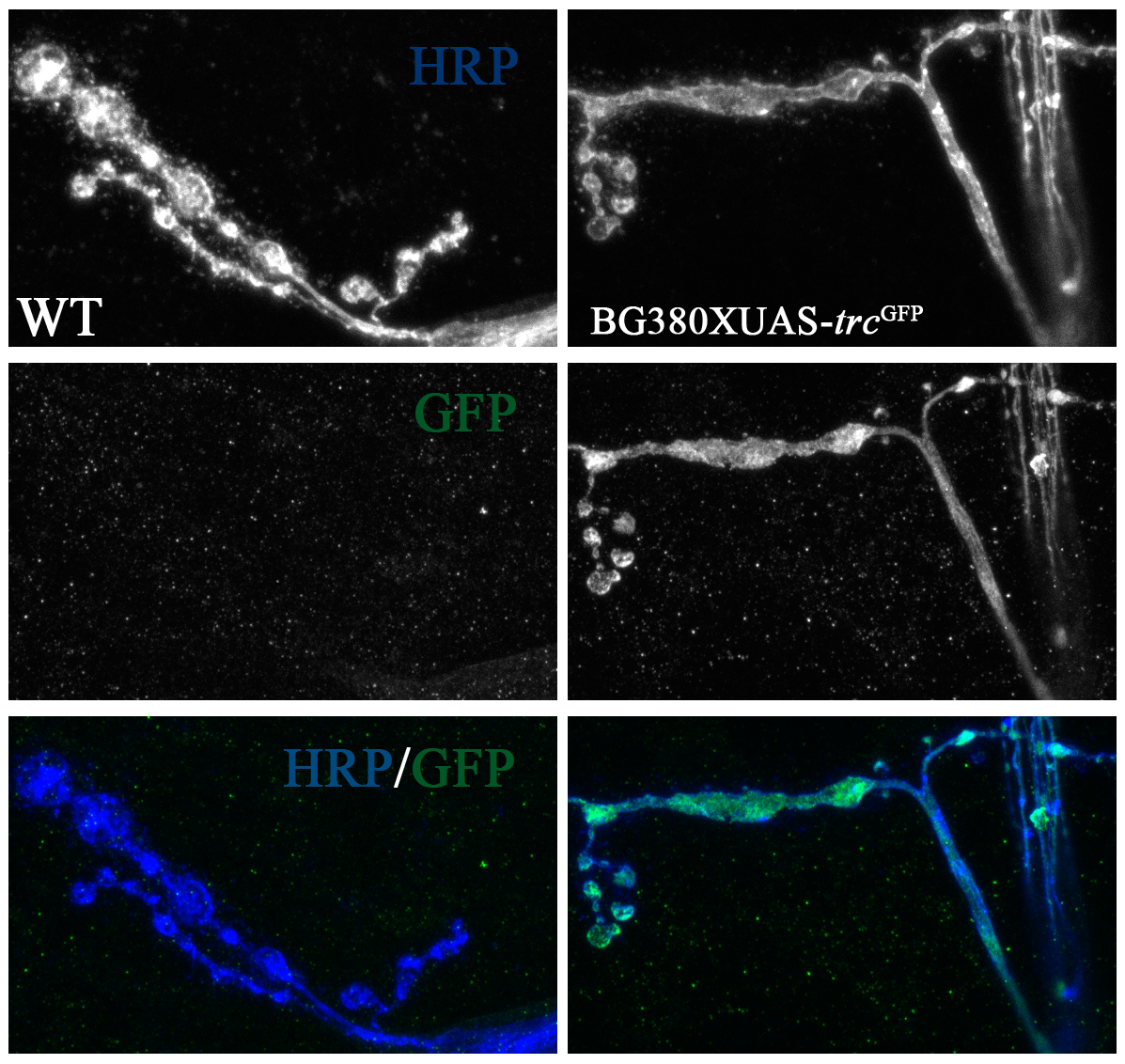

Supplement: S5 Fig — Representative images from muscle 4 of WT and flies overexpressing GFP-tagged Trc when expressed using neuronal driver (BG.380 GAL4). The preparation was stained using antibodies against GFP and HRP. (TIF) [file pone.0138188.s005.tif]
