# Supplementary material for: Tricornered Kinase Regulates Synapse Development by Regulating the Levels of Wiskott-Aldrich Syndrome Protein
Source: PLoS One. 2015 Sep 22;10(9):e0138188. doi: 10.1371/journal.pone.0138188 (PMC4578898; doi:10.1371/journal.pone.0138188)
Supplement: S1 File — (DOCX) [file pone.0138188.s006.docx]

**Supplementary Methods:**

Quantification of branching was performed manually. A branch was any outgrowth that had one or more boutons emanating out of the main branch or a secondary branch. Simple neurite tracer tool plugin in Image J was used to trace the NMJ synapses. Anti-GFP antibodies from Santacruz Biotechnology Inc. (SC-390394) were used to label trc-GFP.
