# Supplementary material for: Tricornered Kinase Regulates Synapse Development by Regulating the Levels of Wiskott-Aldrich Syndrome Protein
Source: PLoS One. 2015 Sep 22;10(9):e0138188. doi: 10.1371/journal.pone.0138188 (PMC4578898; doi:10.1371/journal.pone.0138188)
Supplement: S1 Table — Table showing the genes that are thought to play a role in mTORC2/Akt pathway. The first column represents the Drosophila homolog screened and the mammalian homolog is in parenthesis. Second column represents the alleles screened in our screen and the third column reports the results from the screen. If the NMJs were altered in any way (more or fewer synaptic boutons) then it is represented in the column as “Yes” or else it is referred to as “No”. (PDF) [file pone.0138188.s007.pdf]

Drosophila Gene Name [Mammalian Homolog]	Alleles	Mutant Phenotype
Salt-inducible kinase 2 [QIK]	P{EP}Sik2^{G366}	No
6-phosphofructo-2-kinase (pfrx-PB) [PFKFB2]	Pfrx^{KG02298}	No
Acinus	Acn^{EY22500}	No
Adherens junction protein p120 [*CTNND2*]	ctn^{MB06056}	No
APS	Aps^{BG00831}	No
Ataxin-1	Atx-1$^{[f01201]}$	No
armadillo (arm-PF) [CTNNB1 (b-Catenin)]	*arm*^{1}	No
bruno-2 (bru-2-PL) [CUGBP1]	*bru-2*$^{[MI05725]}$	No
buffy-PA [Bcl-xL]	*Buffy*^{EY11259}	No
Ca2+-channel-protein-β-subunit (CG6320) [CACNB2]	l(2)*CA2*^{HG1}	No
casein kinase ialpha, isoform D. [CK1-D]	*CkIα*^{G0492}	No
cass [Bad]	*cass*$^{2L-5}$	No
cdc2c-PA [CDK2]	*Cdk2*^{KK112530}	No
centaurin beta 1A (cenB1A-PA) [CENTB1 (ACAP1)]	CenB1A^{GS6002}	No
CG10673 [PRPK]	Prpk^{GD7059}	No
CG17184-PA [*Arfaptin 2*]	CG17184^{EY11874}	Yes
CG4045-PA [METTL1]	CG4045^{GD11201}	No
CG43143-PE [NuaK1]	CG43143^{f03049}	No
CG7033 [CCT2]	CG7033^{GD5201}	No
CG9705-PA [CaRHSP1]	CG9705^{KG07795}	No
Cheerio [*FLNC*]	cher^{G9093}, cher^{BG02734}	No
chico-PB [IRS-1]	chico^{170B}	No
dauther of sevenless(dos-PA) [Gab2]	Dmel\dos^{R31}	No
death caspase-1 (Dcp-1-PA) [Caspase-9]	Dcp-1^{02132}pita^{02132}	No
degringolade (dgrn-PA) [BRCA1]	dgrn^{EY09862}	No
Didum [*MYO5A*]	didum^{KG04384}	No
enhancer of decapping 3 (EDC3 PB) [EDC3]	Edc^{3KG01392}	No
enhancer of zeste (E(z)-PB) [Ezh2]	w^{zm,} w^{DZL}	No
ERR (Estrogen-releated receptor) [AR (Androgen Receptor)]	p{EP}ERR^{G4389}	No
eukaryotic initiation factor 4B (elF-4B-PB) [eIF4B]	P{Trip.HMS04503} (RNAi)	No
fork head (fkh-PC) [FOXA2]	fkh^{6}	No
foxhead box, sub-group O	foxo[Delta 94]	No

Girdin-PC [Girdin]	$Girdin^{KG07727}$	No
grain (grn-PB) [GATA-1]	grn^{H10}	No
grapes (grp-PA) [Chk1]	grp^{06034}	No
held out wings [*who*]	how^{24B}	No
Helix loop helix protein 3B (HLH3B-PA) [TAL-1]	$HLH3B^{GD4356}$	No
Heme Oxygense [HMOX1]	No stocks available	N/A
Heterogeneous nuclear ribonucleoprotein at 87F (Hrb87F-PD) [hnRNP A1]	$Hrb87F^{KG02089}$	No
Hippo [*MST1*]	hpo^{G3315}	No
His2B:CG33870-PA [H2B]	$His2B:CG17949^{KG04881}$	
(hop-PA) [TTC3]	hop^{27}	No
Hormone receptor-like in 38 (Hr38) [Nur77]	$Hr38^{02206}$	No
HtrA2	$HtrA2^{f03785}$	No
huntingtin (htt)	htt^{f05417}	No
Inhibitor of apoptosis 2 (Iap-2PB) [XIAP]	$Diap2^{GD1466}$	No
IκB kinase-like 2 (ik2-PB) [IKK-a]	$ik2^{1}$	No
Inositol 1,4,5,-tris-phosphate receptor [IP3R1]	$Ip3K1^{EY09888}$	No
Lamin	Lam^{A25},Lam^{04643}	No
LIM-kinase1	$LIMK1^{EY08757}$	Yes
Lobe (L-PA) [PRAS40 (Akt1S1)]	$PRAS40^{EY01539}$	No
MAD1	$Mad1^{GD9715}$	No
megator (mTOR)	$Mtor^{k03905}$	No
MAP kinase kinase 4 (mkk4) [SEK1]	$Mkk4^{GD13804}$	No
Moesin (moe-PL) [Ezrin]	Moe^{G0323}	No
Misexpression suppressor of KSR 2 (MESK2-PB) [Ndrg2]	$MESK2^{01467}$	No
Mtl	$Rac1^{J11}$, $Rac2^{\Delta}$, Mtl^{Δ}	N.D.
mushroom-body expressed (mub-PG) [hnRNP E1]	mbd^{1}	No
mutagen-sensitive 101 (mus101 PA) [TOPBP1]	$mei\text{-}9^{b}$	No
Myt1	$Myt1^{GD10889}$	No
nejire-PB [CBP]	CBP^{EP1201}	No
nejire (nej-PB) [p300]	nej^{3}	No
NMDA receptor 2 (Nmdar2-PC) [NMDAR2C]	$Nmdar2^{GD1621}$	No
Phosphodiesterase 1c [PDE3B]	Pde1c[c04487]	No
plexin A [*ron*]	$plexA^{EY16548}$,	No
polar granule component [PGC-1]	$pgc^{EY09338}$	No

pole hole (phl) [Raf1 (c-Raf)]	phl^{7}	No
Pollux [*AS160*]	plx^{MI02460}, plx^{MB01981}	No
Plenty of SH3s (POSH)	POSH^{EP1206}, POSH^{k15815}	No
Programmed cell death 4 ortholog [PDCD4]	Pdcd4^{BG00494}	No
protein kinase at 92B [ASK1]	Pk92B^{GD11408}	No
Rac 1	Rac1^{J11}	No
Rac 2	Rac1^{J11}, Rac2$^{\Delta}$	No
Ran binding protein 3 [RANBP3]	RanBP3^{KG08193}	No
Ribosomal protein S6 [S6]	mRpS6^{GD8514}	No
Sallimus [*palladin*]	sls^{1}, sls^{rL182}	No
Skp2-PA [Skp2]	Skp2^{GD5142}	No
slipper [*MLK3*]	slpr^{MB03655}	No
sloppy paired 2 (slp2-PA) [FOXG1]	slp1^{2}	No
small wing (sl-PA) [PLCg1]	sl^{2}	No
Sno oncogene (Snoo-PD) [SKI]	Snoo^{GD12126}	No
Src oncogene at 42A [SH3BP4]	Src42A^{k10115}	No
ssb	Ssb-c31a^{e02272}	No
string (stg-PA) [Cdc25B]	stg^{GS3120}	No
TBP-related factor (trf) [TRF1]	Trf^{1}	No
Tetraspanin [Peripherin]	Tsp42Ea^{KG05090}	No
TER94 [VCP]	TER94^{03775}	No
thread [MDM2]	Diap1	No
Tricornered [*NDR*]	trc^{1}	Yes
Twinstar [*cofilin*]	tsr^{1}, tsr^{N96A}	No
UBPY ortholog (Ubpy-PA) [USP8]	Usp8^{HMS01898}	No
yorkie [YAP1]	yki^{B5}	No
(wnk-PD) [WNK1]	Wnk^{EY10165}	No
Zyxin	Zyxin RNAi	No
